# The risk factors for developing exposure keratopathy in ICU patients

**DOI:** 10.1186/2197-425X-3-S1-A731

**Published:** 2015-10-01

**Authors:** O Kousha, Z Kousha, J Paddle

**Affiliations:** Royal Cornwall Hospital NHS Trust, Truro, United Kingdom; University College London, Faculty of Medical Sciences, London, United Kingdom

## Introduction

Intensive care unit (ICU) patients are at increased risk of developing exposure keratopathy (EK) due to intubation, sedation, paralysis and metabolic disturbance. The factors lead to reduced venous return from the eyes, impairment of the blink reflex, loss of the tone of the orbicularis oculi muscles and dysfunction of the corneal healing. EK can lead to short- and long-term visual impairment.

## Objectives

To ascertain the risk factors for the development of EK in the whole ICU population.

## Methods

A prospective cohort study of all patients admitted consecutively over a four months period, from 24/11/2014 to 31/03/2015. A total of 257 patients were included and 17 were excluded (2 patients refused consent, 4 patients were too agitated, 3 patients had pre-existing ocular surface disease and 8 patients were below the age of 16). Every patient was assessed everyday using a standardised pro forma with a portable slit lamp. A total of 2712 eye assessments were carried out.

## Results

The incidence of EK in mechanically ventilated patients was 54.3% compared to 5.1% in patients receiving non-invasive ventilation or no ventilatory support (*p < 0.001*). Relative risk was 10.6 (95% confidence Interval (CI) 5.5 to 20.7). A logistic regression model was constructed to calculate adjusted odds ratios (OR) for various factors (Table 1). Mechanical ventilation and lagophthalmos were identified as the main risk factor for developing EK with lagophthalmos having the largest effect. Among mechanically ventilated patients who received less than 3 times daily eye care, the incidence of EK was 64.5% compared to 11.5% in the patients receiving eye care 3 times a day or more (*p < 0.001*). Relative risk was 5.6 (95% CI 3.8 to 7.7).

**Table 1:** Adjusted OR of developing EK for various factors

*Risk Factors*	*OR (95% CI)*	*p-value*
Male Gender	1.1 (0.5 - 2.7)	0.733
Age^a^	1.0 (1.0 - 1.1)	0.191
APACHE II score^b^	1.1 (1.0 - 1.1)	0.104
Mechanical ventilation	**6.8 (3.2 - 8.0)**	**0.028**
Sedation	1.2 (0.3 - 7.7)	0.068
Lagophthalmos	**32.5 (15.3 - 45.1)**	**< 0.001**

^*a*^One year increase in age

^*b*^One point increase in APACHE II score

## Conclusions

EK is a common but preventable condition in ICU patients with the major risks being mechanical ventilation and lagophthalmos. However, prevention and treatment strategies can be developed to identify the patients at risk, prevent the development of EK and, if EK develops, to treat EK.

